# Supplementary material for: Prioritizing interventions for cholera control in Kenya, 2015–2020
Source: PLoS Negl Trop Dis. 2023 May 17;17(5):e0010928. doi: 10.1371/journal.pntd.0010928 (PMC10228803; doi:10.1371/journal.pntd.0010928)
Supplement: S1 Text — Fig A in S1 Text: Data Cleaning Flow Chart. Fig B in S1 Text: Total annual cholera cases by sub-counties, Kenya, 2015–2020. Fig C in S1 Text: Scatterplot for Mean Annual Incidence versus persistence (Sensitivity Analysis). Fig D in S1 Text. Priority intervention areas at the sub-county scale, using a different location name cleaning methodology–Sensitivity Analysis (MAI threshold: 90 percentile– 31.4 per 100,000, Persistence threshold: median– 5.45%). Fig E in S1 Text: Sensitivity analysis for priority intervention areas at the sub-county scale after assuming different thresholds for mean annual incidence. The persistence threshold remained constant at the median value of 2.88%. Table A in S1 Text: Number of missing subcounty observations by county and year. Table B in S1 Text. Population and number of sub-counties under each hotspot level (sensitivity analysis) based on MAI threshold: 90 percentile– 39 per 100,000, Persistence threshold: median– 2.88%. Table C in S1 Text. Hotspot sensitivity transition matrix. Presents sub-county hotspot classification change based on data cleaning. The Clean Data Hotspot are records that had insufficient data to categorize the case, and as a result, were exluded from the Analysis. The Sensitivity Analysis Hotspot takes the same record with insufficient data and uses extrapolation to assign the sub-county and county information. Table D in S1 Text. Table provides % population residing in a defined hotspot area based on MAI threshold. Table E in S1 Text. Subcounty by Priority. Table F in S1 Text. Test Summary by County by Year. (DOCX) [file pntd.0010928.s001.docx]

S1 Text: Supporting Information

Fig A: Data Cleaning Flow Chart

Table A: Number of missing subcounty observations by county and year

|  | **2015** | **2016** | **2017** | **2018** | **2019** | **2020** |
| --- | --- | --- | --- | --- | --- | --- |
| **Busia** | 0 | 0 | 30 | 42 | 0 | 0 |
| **Elgeyo-Marakwet** | 0 | 0 | 0 | 0 | 0 | 0 |
| **Embu** | 142 | 0 | 9 | 0 | 6 | 0 |
| **Garissa** | 119 | 118 | 646 | 52 | 887 | 0 |
| **Homa Bay** | 25 | 0 | 0 | 0 | 0 | 0 |
| **Isiolo** | 0 | 0 | 0 | 16 | 0 | 0 |
| **Kajiado** | 0 | 41 | 0 | 0 | 78 | 0 |
| **Kiambu** | 42 | 0 | 0 | 7 | 0 | 0 |
| **Kilifi** | 59 | 0 | 17 | 0 | 0 | 0 |
| **Kirinyaga** | 260 | 0 | 40 | 3 | 0 | 0 |
| **Kisumu** | 1 | 0 | 0 | 0 | 2 | 0 |
| **Kwale** | 0 | 0 | 9 | 0 | 0 | 0 |
| **Machakos** | 26 | 0 | 9 | 23 | 401 | 0 |
| **Makueni** | 0 | 0 | 0 | 0 | 0 | 0 |
| **Mandera** | 0 | 611 | 0 | 0 | 352 | 0 |
| **Marsabit** | 20 | 0 | 0 | 0 | 0 | 0 |
| **Meru** | 0 | 3 | 0 | 0 | 0 | 0 |
| **Migori** | 69 | 0 | 0 | 0 | 0 | 0 |
| **Mombasa** | 2 | 0 | 0 | 1 | 1 | 0 |
| **Murang'a** | 193 | 0 | 0 | 7 | 0 | 1 |
| **Nairobi** | 232 | 6 | 292 | 3 | 423 | 0 |
| **Nakuru** | 1 | 20 | 3 | 10 | 0 | 0 |
| **Nandi** | 0 | 0 | 0 | 0 | 0 | 0 |
| **Narok** | 0 | 0 | 0 | 0 | 14 | 0 |
| **Siaya** | 13 | 0 | 0 | 6 | 0 | 0 |
| **Tana River** | 0 | 1 | 0 | 40 | 0 | 0 |
| **Tharaka-Nithi** | 0 | 276 | 33 | 280 | 0 | 0 |
| **Trans Nzoia** | 0 | 0 | 0 | 27 | 0 | 0 |
| **Turkana** | 0 | 0 | 13 | 4 | 0 | 0 |
| **Vihiga** | 0 | 29 | 0 | 0 | 0 | 0 |
| **Wajir** | 0 | 0 | 0 | 0 | 0 | 0 |
| **West Pokot** | 0 | 0 | 0 | 161 | 0 | 0 |

Fig B: Total annual cholera cases by sub-counties, Kenya, 2015-2020


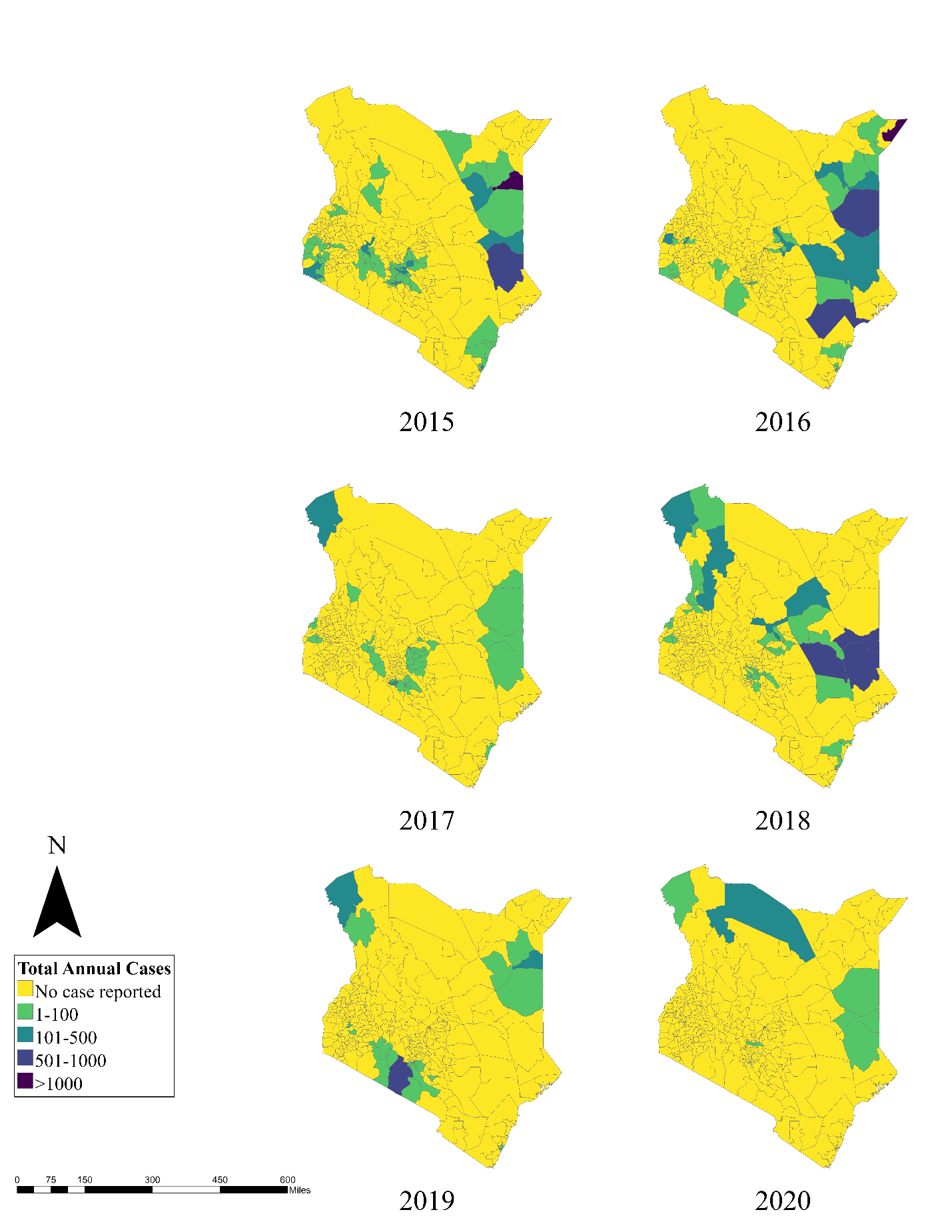


Fig C: Scatterplot for Mean Annual Incidence versus persistence (Sensitivity Analysis)


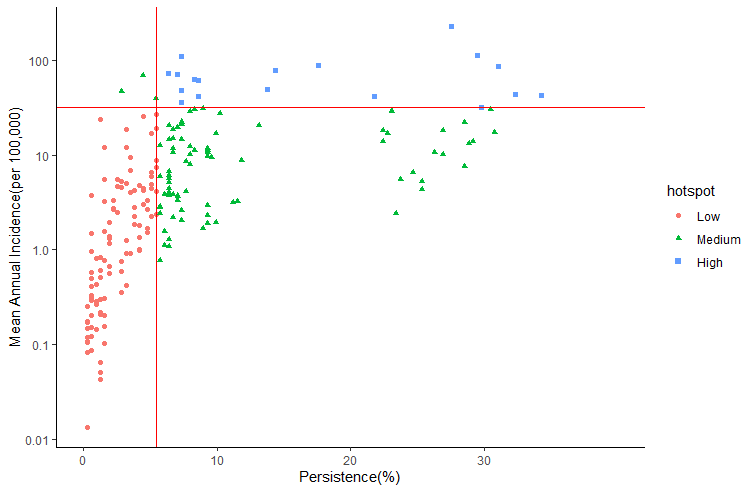


Fig D: Priority intervention areas at the sub-county scale, using a different location name cleaning methodology – Sensitivity Analysis (MAI threshold: 90 percentile – 31.4 per 100,000, Persistence threshold: median – 5.45%)


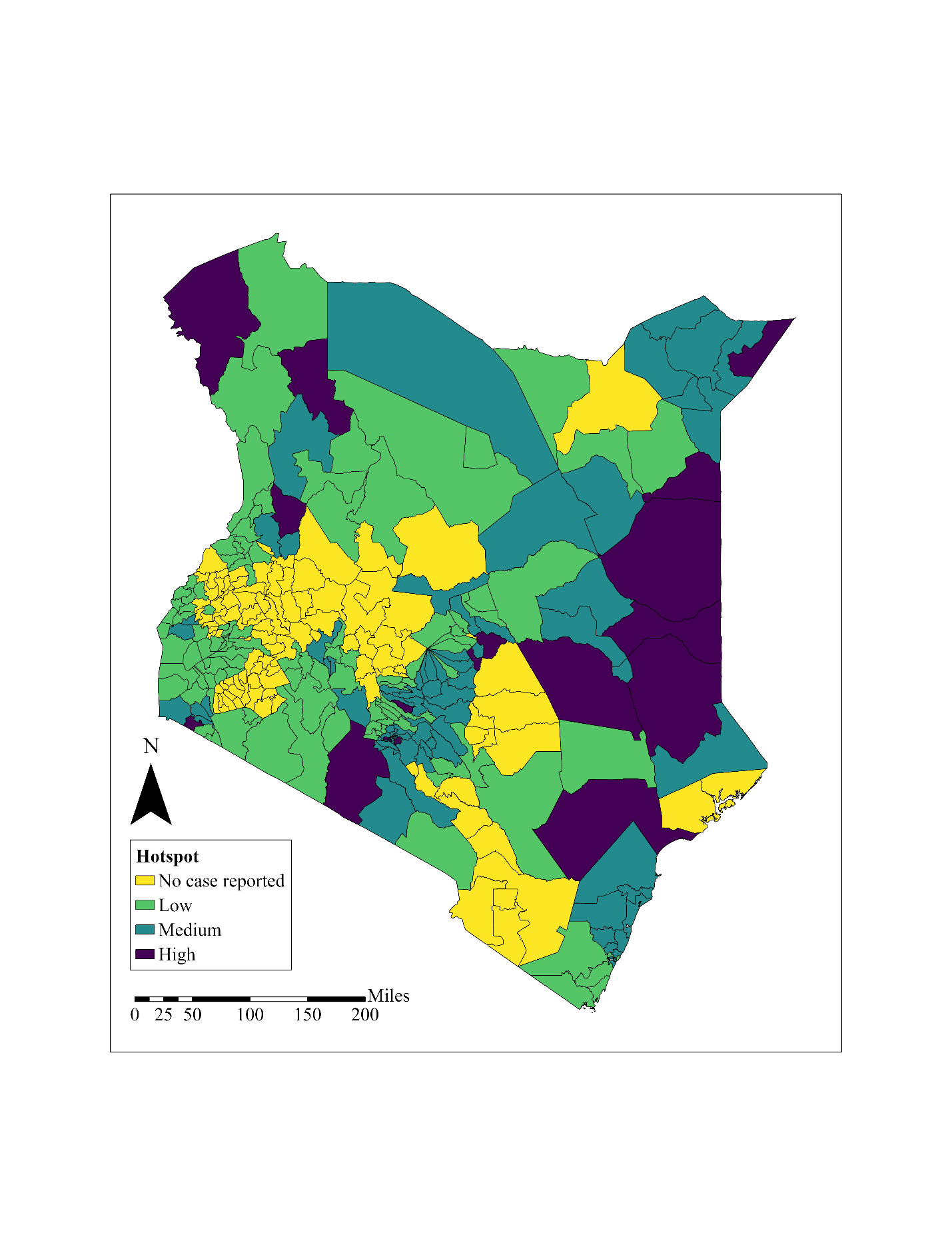


Fig E: Sensitivity analysis for priority intervention areas at the sub-county scale after assuming different thresholds for mean annual incidence. The persistence threshold remained constant at the median value of 2.88%.


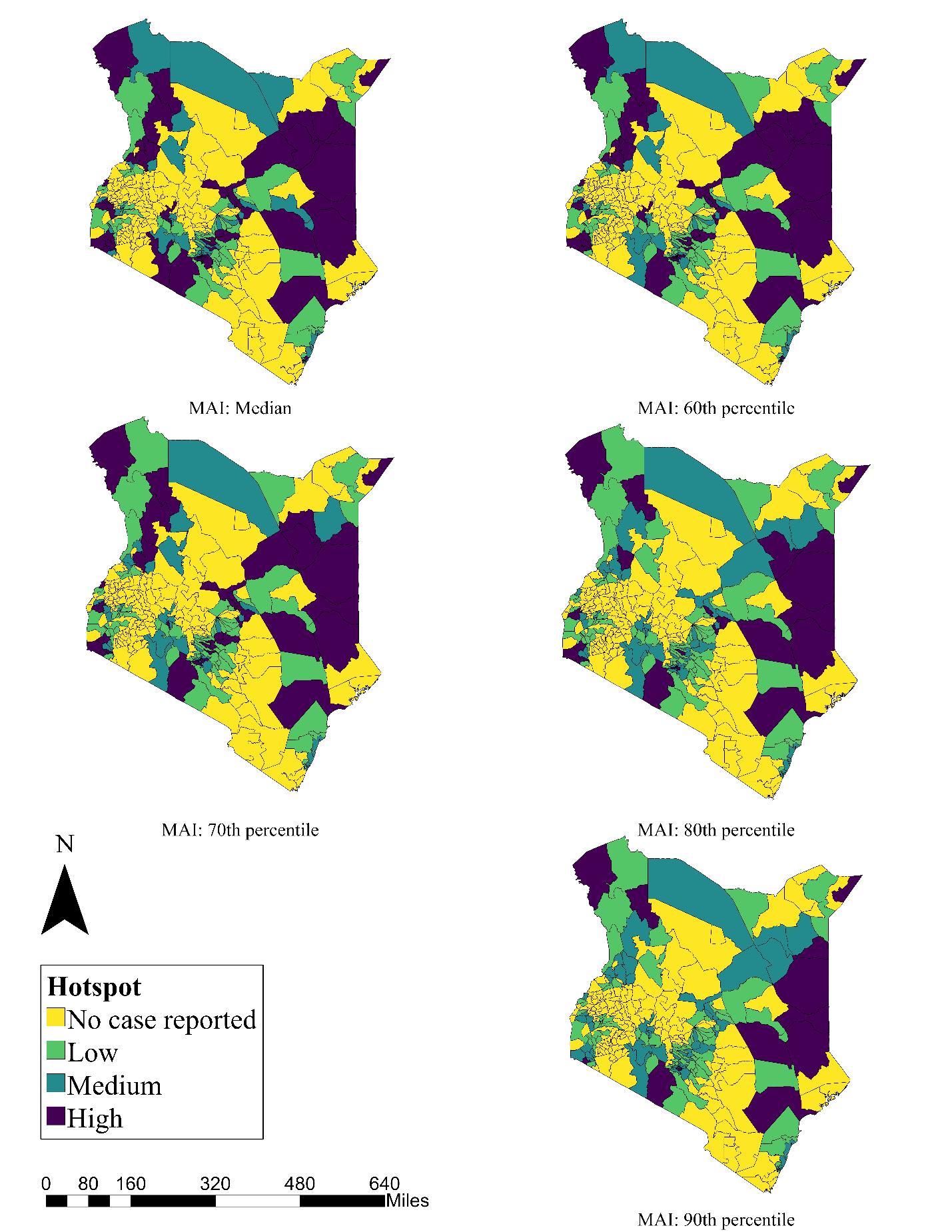


Table B. Population and number of sub-counties under each hotspot level (sensitivity analysis) based on MAI threshold: 90 percentile – 39 per 100,000, Persistence threshold: median – 2.88%

| **Hotspot Level** | **Population** | **Percentage of population** | **Number of sub-counties** | **Population Weighted Average**  **MAI (cases per 100,000):**  **Mean (Min, Max)** |
| --- | --- | --- | --- | --- |
| High | 3643486 | 7.2 | 18 | 71.48 (31.47, 221.55) |
| Medium | 15920789 | 31.4 | 80 | 10.52 (0.78, 68.22) |
| Low | 16247960 | 32.0 | 104 | 3.24 (0.01, 26.65) |
| No case reported | 14934673 | 29.4 | 99 | 0 |

Table C: Hotspot sensitivity transition matrix. Presents sub-county hotspot classification change based on data cleaning. The Clean Data Hotspot are records that had insufficient data to categorize the case, and as a result, were exluded from the Analysis. The Sensitivity Analysis Hotspot takes the same record with insufficient data and uses extrapolation to assign the sub-county and county information.

|  | | Sensitivity Analysis Hotspot (extrapolated data) | | | |
| --- | --- | --- | --- | --- | --- |
|  |  | No case reported | Low | Medium | High |
| Clean Data Hotspot | No case reported | 99 | 44 | 8 | 0 |
|  | Low | 0 | 46 | 29 | 1 |
|  | Medium | 0 | 14 | 43 | 4 |
|  | High | 0 | 0 | 0 | 13 |

Table D: Table provides % population residing in a defined hotspot area based on MAI threshold

|  | | Population (%) under each hotspot level | | | |
| --- | --- | --- | --- | --- | --- |
|  |  | No case reported | Low | Medium | High |
| MAI threshold | Median | 23041691(45) | 9964083(20) | 6786915(13) | 10954219(22) |
|  | 60^th^ percentile | 23041691(45) | 11847535(23) | 5531734(11) | 10325949(21) |
|  | 70^th^ percentile | 23041691(45) | 12150050(24) | 7742844(15) | 7812323(16) |
|  | 80^th^ percentile | 23041691(45) | 12353232(24) | 9992229(20) | 5359756(11) |
|  | 90^th^ percentile | 23041691(45) | 12559717(25) | 12124262(24) | 3021238(6) |

Table E: Subcounty by Priority

| **Subcounty** | **County** | **Priority** |
| --- | --- | --- |
| Bura | Tana River | High |
| Daadab | Garissa | High |
| Embakasi East | Nairobi | High |
| Fafi | Garissa | High |
| Garsen | Tana River | High |
| Kajiado West | Kajiado | High |
| Mandera East | Mandera | High |
| Marakwet East | Elgeyo-Marakwet | High |
| North Horr | Marsabit | High |
| Starehe | Nairobi | High |
| Suna West | Migori | High |
| Turkana Central | Turkana | High |
| Turkana West | Turkana | High |
| Wajir East | Wajir | High |
| Wajir South | Wajir | High |
| Aldai | Nandi | Low |
| Balambala | Garissa | Low |
| Bondo | Siaya | Low |
| Buuri | Meru | Low |
| Cherangany | Trans Nzoia | Low |
| Chuka/Igambang'Ombe | Tharaka-Nithi | Low |
| Galole | Tana River | Low |
| Ganze | Kilifi | Low |
| Garissa Township | Garissa | Low |
| Gatanga | Murang'a | Low |
| Gatundu North | Kiambu | Low |
| Gilgil | Nakuru | Low |
| Githunguri | Kiambu | Low |
| Hamisi | Vihiga | Low |
| Homa Bay Town | Homa Bay | Low |
| Igembe Central | Meru | Low |
| Igembe South | Tharaka-Nithi | Low |
| Igembe South | Meru | Low |
| Isiolo South | Isiolo | Low |
| Jomvu | Mombasa | Low |
| Kabete | Kiambu | Low |
| Kacheliba | West Pokot | Low |
| Kaiti | Makueni | Low |
| Kajiado Central | Kajiado | Low |
| Kajiado East | Kajiado | Low |
| Kajiado North | Kajiado | Low |
| Kangema | Murang'a | Low |
| Kangundo | Machakos | Low |
| Karachuonyo | Homa Bay | Low |
| Kasipul | Homa Bay | Low |
| Kathiani | Machakos | Low |
| Kigumo | Murang'a | Low |
| Kiharu | Murang'a | Low |
| Kikuyu | Kiambu | Low |
| Kiminini | Trans Nzoia | Low |
| Kirinyaga Central | Kirinyaga | Low |
| Kisumu Central | Kisumu | Low |
| Kisumu East | Kisumu | Low |
| Kisumu West | Kisumu | Low |
| Kuria West | Migori | Low |
| Loima | Turkana | Low |
| Maara | Tharaka-Nithi | Low |
| Machakos Town | Machakos | Low |
| Magarini | Kilifi | Low |
| Malindi | Kilifi | Low |
| Mandera North | Mandera | Low |
| Mandera South | Mandera | Low |
| Manyatta | Embu | Low |
| Masinga | Machakos | Low |
| Matungulu | Machakos | Low |
| Mbeere North | Embu | Low |
| Mbita | Homa Bay | Low |
| Molo | Nakuru | Low |
| Moyale | Marsabit | Low |
| Muhoroni | Kisumu | Low |
| Mwala | Machakos | Low |
| Nambale | Busia | Low |
| Narok East | Narok | Low |
| Ndia | Kirinyaga | Low |
| Njoro | Nakuru | Low |
| North Imenti | Meru | Low |
| Nyakach | Kisumu | Low |
| Rabai | Kilifi | Low |
| Rangwe | Homa Bay | Low |
| Rongo | Migori | Low |
| Runyenjes | Embu | Low |
| Sabatia | Vihiga | Low |
| Teso South | Busia | Low |
| Turkana East | Turkana | Low |
| Turkana North | Turkana | Low |
| Ugenya | Siaya | Low |
| Alego Usonga | Siaya | Medium |
| Awendo | Migori | Medium |
| Bahati | Nakuru | Medium |
| Changamwe | Mombasa | Medium |
| Dagoretti North | Nairobi | Medium |
| Dagoretti South | Nairobi | Medium |
| Eldas | Wajir | Medium |
| Embakasi Central | Nairobi | Medium |
| Embakasi South | Nairobi | Medium |
| Embakasi West | Nairobi | Medium |
| Gatundu South | Kiambu | Medium |
| Gem | Siaya | Medium |
| Gichugu | Kirinyaga | Medium |
| Isiolo North | Isiolo | Medium |
| Juja | Kiambu | Medium |
| Kamukunji | Nairobi | Medium |
| Kandara | Murang'a | Medium |
| Kapenguria | West Pokot | Medium |
| Kasarani | Nairobi | Medium |
| Kiambaa | Kiambu | Medium |
| Kiambu | Kiambu | Medium |
| Kibra | Nairobi | Medium |
| Kilifi North | Kilifi | Medium |
| Kilifi South | Kilifi | Medium |
| Kisauni | Mombasa | Medium |
| Kwanza | Trans Nzoia | Medium |
| Langata | Nairobi | Medium |
| Likoni | Mombasa | Medium |
| Makadara | Nairobi | Medium |
| Maragwa | Murang'a | Medium |
| Mathare | Nairobi | Medium |
| Mavoko | Machakos | Medium |
| Mbeere South | Embu | Medium |
| Mvita | Mombasa | Medium |
| Mwea | Kirinyaga | Medium |
| Naivasha | Nakuru | Medium |
| Nakuru Town East | Nakuru | Medium |
| Nakuru Town West | Nakuru | Medium |
| Narok North | Narok | Medium |
| Narok South | Narok | Medium |
| Ndhiwa | Homa Bay | Medium |
| Nyali | Mombasa | Medium |
| Nyatike | Migori | Medium |
| Pokot South | West Pokot | Medium |
| Rongai | Nakuru | Medium |
| Roysambu | Nairobi | Medium |
| Ruaraka | Nairobi | Medium |
| Ruiru | Kiambu | Medium |
| Saboti | Trans Nzoia | Medium |
| Seme | Kisumu | Medium |
| Sigor | West Pokot | Medium |
| Suna East | Migori | Medium |
| Tarbaj | Wajir | Medium |
| Teso North | Busia | Medium |
| Tharaka | Tharaka-Nithi | Medium |
| Thika Town | Kiambu | Medium |
| Tigania East | Meru | Medium |
| Tigania West | Meru | Medium |
| Turkana South | Turkana | Medium |
| Ugunja | Siaya | Medium |
| Uriri | Migori | Medium |
| Wajir West | Wajir | Medium |
| Westlands | Nairobi | Medium |
| Yatta | Machakos | Medium |
| Mogotio Urban | Baringo | No case reported |
| Ainabkoi | Uasin Gishu | No case reported |
| Ainamoi | Kericho | No case reported |
| Banissa | Mandera | No case reported |
| Baringo Central | Baringo | No case reported |
| Baringo North | Baringo | No case reported |
| Baringo South | Baringo | No case reported |
| Belgut | Kericho | No case reported |
| Bobasi | Kisii | No case reported |
| Bomachoge Borabu | Kisii | No case reported |
| Bomachoge Chache | Kisii | No case reported |
| Bomet Central | Bomet | No case reported |
| Bomet East | Bomet | No case reported |
| Bonchari | Kisii | No case reported |
| Borabu | Nyamira | No case reported |
| Budalangi | Busia | No case reported |
| Bumula | Bungoma | No case reported |
| Bureti | Kericho | No case reported |
| Butere | Kakamega | No case reported |
| Butula | Busia | No case reported |
| Central Imenti | Meru | No case reported |
| Chepalungu | Bomet | No case reported |
| Chesumei | Nandi | No case reported |
| Eldama Ravine | Baringo | No case reported |
| Embakasi North | Nairobi | No case reported |
| Emgwen | Nandi | No case reported |
| Emuhaya | Vihiga | No case reported |
| Emurua Dikirr | Narok | No case reported |
| Endebess | Trans Nzoia | No case reported |
| Funyula | Busia | No case reported |
| Igembe North | Meru | No case reported |
| Ijara | Garissa | No case reported |
| Ikolomani | Kakamega | No case reported |
| Kabondo Kasipul | Homa Bay | No case reported |
| Kabuchai | Bungoma | No case reported |
| Kajiado South | Kajiado | No case reported |
| Kaloleni | Kilifi | No case reported |
| Kanduyi | Bungoma | No case reported |
| Kapseret | Uasin Gishu | No case reported |
| Keiyo North | Elgeyo-Marakwet | No case reported |
| Keiyo South | Elgeyo-Marakwet | No case reported |
| Kesses | Uasin Gishu | No case reported |
| Khwisero | Kakamega | No case reported |
| Kibwezi East | Makueni | No case reported |
| Kibwezi West | Makueni | No case reported |
| Kieni | Nyeri | No case reported |
| Kilgoris | Narok | No case reported |
| Kilome | Makueni | No case reported |
| Kimilili | Bungoma | No case reported |
| Kinango | Kwale | No case reported |
| Kinangop | Nyandarua | No case reported |
| Kipipiri | Nyandarua | No case reported |
| Kipkelion East | Kericho | No case reported |
| Kipkelion West | Kericho | No case reported |
| Kitui Central | Kitui | No case reported |
| Kitui East | Kitui | No case reported |
| Kitui Rural | Kitui | No case reported |
| Kitui South | Kitui | No case reported |
| Kitui West | Kitui | No case reported |
| Kitutu Chache North | Kisii | No case reported |
| Kitutu Chache South | Kisii | No case reported |
| Kitutu Masaba | Nyamira | No case reported |
| Konoin | Bomet | No case reported |
| Kuresoi North | Nakuru | No case reported |
| Kuresoi South | Nakuru | No case reported |
| Kuria East | Migori | No case reported |
| Lafey | Mandera | No case reported |
| Lagdera | Garissa | No case reported |
| Laikipia East | Laikipia | No case reported |
| Laikipia North | Laikipia | No case reported |
| Laikipia West | Laikipia | No case reported |
| Laisamis | Marsabit | No case reported |
| Lamu East | Lamu | No case reported |
| Lamu West | Lamu | No case reported |
| Lari | Kiambu | No case reported |
| Likuyani | Bungoma | No case reported |
| Limuru | Kiambu | No case reported |
| Luanda | Vihiga | No case reported |
| Lugari | Bungoma | No case reported |
| Lugari | Kakamega | No case reported |
| Lungalunga | Kwale | No case reported |
| Lurambi | Kakamega | No case reported |
| Makueni | Makueni | No case reported |
| Malava | Kakamega | No case reported |
| Mandera West | Mandera | No case reported |
| Marakwet West | Elgeyo-Marakwet | No case reported |
| Matayos | Busia | No case reported |
| Mathioya | Murang'a | No case reported |
| Mathira | Nyeri | No case reported |
| Matuga | Kwale | No case reported |
| Matungu | Kakamega | No case reported |
| Mbooni | Makueni | No case reported |
| Mogotio | Baringo | No case reported |
| Moiben | Uasin Gishu | No case reported |
| Mosop | Nandi | No case reported |
| Msambweni | Kwale | No case reported |
| Mt. Elgon | Bungoma | No case reported |
| Mukurweini | Nyeri | No case reported |
| Mumias East | Kakamega | No case reported |
| Mumias West | Kakamega | No case reported |
| Mwatate | Taita Taveta | No case reported |
| Mwingi Central | Kitui | No case reported |
| Mwingi North | Kitui | No case reported |
| Mwingi West | Kitui | No case reported |
| Nandi Hills | Nandi | No case reported |
| Narok West | Narok | No case reported |
| Navakholo | Kakamega | No case reported |
| Ndaragwa | Nyandarua | No case reported |
| North Mugirango | Nyamira | No case reported |
| Nyando | Kisumu | No case reported |
| Nyaribari Chache | Kisii | No case reported |
| Nyaribari Masaba | Kisii | No case reported |
| Nyeri Town | Nyeri | No case reported |
| Ol Jorok | Nyandarua | No case reported |
| Ol Kalou | Nyandarua | No case reported |
| Othaya | Nyeri | No case reported |
| Rarieda | Siaya | No case reported |
| Saku | Marsabit | No case reported |
| Samburu East | Samburu | No case reported |
| Samburu North | Samburu | No case reported |
| Samburu West | Samburu | No case reported |
| Shinyalu | Kakamega | No case reported |
| Sigowet/Soin | Kericho | No case reported |
| Sirisia | Bungoma | No case reported |
| Sotik | Bomet | No case reported |
| South Imenti | Meru | No case reported |
| South Mugirango | Kisii | No case reported |
| Soy | Uasin Gishu | No case reported |
| Suba | Homa Bay | No case reported |
| Subukia | Nakuru | No case reported |
| Taveta | Taita Taveta | No case reported |
| Tetu | Nyeri | No case reported |
| Tiaty | Baringo | No case reported |
| Tinderet | Nandi | No case reported |
| Tongaren | Bungoma | No case reported |
| Turbo | Uasin Gishu | No case reported |
| unknown 1 | Mandera | No case reported |
| unknown 2 | Turkana | No case reported |
| unknown 3 | West Pokot | No case reported |
| unknown 4 | Trans Nzoia | No case reported |
| unknown 5 | Meru | No case reported |
| unknown 6 | Homa Bay | No case reported |
| unknown 7 | Machakos | No case reported |
| unknown 8 | West Pokot | No case reported |
| Vihiga | Vihiga | No case reported |
| Voi | Taita Taveta | No case reported |
| Wajir North | Wajir | No case reported |
| Webute West | Bungoma | No case reported |
| Webuye East | Bungoma | No case reported |
| West Mugirango | Nyamira | No case reported |
| Wundanyi | Taita Taveta | No case reported |

Table F: Test Summary by County by Year

| **County** | **Year** | **Total (n)** | **RDT Test** | **RDT Positive** | **Culture Test** | **Culture Positive** |
| --- | --- | --- | --- | --- | --- | --- |
| Embu | 2015 | 231 | 31 | 24 | 30 | 11 |
| Garissa | 2015 | 1088 | 0 | 0 | 28 | 27 |
| Homa Bay | 2015 | 358 | 0 | 0 | 94 | 36 |
| Kiambu | 2015 | 291 | 0 | 0 | 101 | 31 |
| Kilifi | 2015 | 100 | 0 | 0 | 100 | 44 |
| Kirinyaga | 2015 | 435 | 0 | 0 | 25 | 9 |
| Kisumu | 2015 | 120 | 9 | 6 | 10 | 10 |
| Machakos | 2015 | 41 | 0 | 0 | 23 | 4 |
| Marsabit | 2015 | 54 | 0 | 0 | 41 | 37 |
| Migori | 2015 | 1020 | 115 | 83 | 24 | 10 |
| Mombasa | 2015 | 293 | 0 | 0 | 109 | 54 |
| Murang'a | 2015 | 735 | 0 | 0 | 103 | 65 |
| Nairobi | 2015 | 1844 | 23 | 20 | 202 | 143 |
| Nakuru | 2015 | 392 | 1 | 1 | 125 | 124 |
| Narok | 2015 | 22 | 10 | 10 | 7 | 3 |
| Siaya | 2015 | 146 | 0 | 0 | 68 | 43 |
| Trans Nzoia | 2015 | 23 | 0 | 0 | 13 | 10 |
| Turkana | 2015 | 46 | 0 | 0 | 7 | 7 |
| Wajir | 2015 | 2307 | 47 | 31 | 354 | 251 |
| Garissa | 2016 | 675 | 0 | 0 | 233 | 115 |
| Homa Bay | 2016 | 1 | 0 | 0 | 0 | 0 |
| Kajiado | 2016 | 42 | 2 | 2 | 3 | 3 |
| Kilifi | 2016 | 7 | 0 | 0 | 6 | 3 |
| Mandera | 2016 | 1785 | 11 | 11 | 227 | 161 |
| Meru | 2016 | 246 | 0 | 0 | 59 | 12 |
| Migori | 2016 | 156 | 7 | 7 | 0 | 0 |
| Mombasa | 2016 | 7 | 0 | 0 | 7 | 2 |
| Nairobi | 2016 | 72 | 0 | 0 | 6 | 6 |
| Nakuru | 2016 | 20 | 0 | 0 | 11 | 11 |
| Nandi | 2016 | 21 | 19 | 19 | 2 | 2 |
| Narok | 2016 | 1 | 1 | 1 | 1 | 1 |
| Siaya | 2016 | 558 | 0 | 0 | 34 | 14 |
| Tana River | 2016 | 767 | 47 | 36 | 13 | 8 |
| Tharaka-Nithi | 2016 | 419 | 0 | 0 | 23 | 10 |
| Vihiga | 2016 | 275 | 0 | 0 | 232 | 20 |
| Wajir | 2016 | 1104 | 0 | 0 | 87 | 81 |
| Busia | 2017 | 92 | 0 | 0 | 0 | 0 |
| Embu | 2017 | 60 | 21 | 18 | 22 | 20 |
| Garissa | 2017 | 657 | 246 | 207 | 410 | 308 |
| Kilifi | 2017 | 29 | 0 | 0 | 28 | 14 |
| Kirinyaga | 2017 | 108 | 11 | 6 | 49 | 33 |
| Kwale | 2017 | 9 | 0 | 0 | 8 | 7 |
| Machakos | 2017 | 68 | 0 | 0 | 21 | 13 |
| Meru | 2017 | 9 | 0 | 0 | 0 | 0 |
| Nairobi | 2017 | 1943 | 631 | 513 | 370 | 251 |
| Nakuru | 2017 | 24 | 14 | 14 | 6 | 6 |
| Siaya | 2017 | 25 | 0 | 0 | 9 | 6 |
| Tharaka-Nithi | 2017 | 43 | 8 | 7 | 3 | 3 |
| Turkana | 2017 | 226 | 168 | 117 | 56 | 37 |
| Wajir | 2017 | 22 | 16 | 3 | 14 | 7 |
| West Pokot | 2017 | 2 | 0 | 0 | 0 | 0 |
| Busia | 2018 | 142 | 0 | 0 | 1 | 1 |
| Elgeyo-Marakwet | 2018 | 320 | 10 | 8 | 1 | 1 |
| Garissa | 2018 | 1359 | 192 | 174 | 76 | 51 |
| Isiolo | 2018 | 149 | 7 | 7 | 3 | 2 |
| Kiambu | 2018 | 80 | 19 | 19 | 26 | 19 |
| Kilifi | 2018 | 4 | 0 | 0 | 4 | 4 |
| Kirinyaga | 2018 | 3 | 0 | 0 | 0 | 0 |
| Machakos | 2018 | 52 | 12 | 12 | 19 | 6 |
| Meru | 2018 | 253 | 1 | 1 | 26 | 3 |
| Mombasa | 2018 | 11 | 0 | 0 | 11 | 4 |
| Murang'a | 2018 | 77 | 0 | 0 | 15 | 15 |
| Nairobi | 2018 | 3 | 1 | 1 | 0 | 0 |
| Nakuru | 2018 | 10 | 0 | 0 | 2 | 2 |
| Siaya | 2018 | 138 | 0 | 0 | 13 | 6 |
| Tana River | 2018 | 584 | 73 | 0 | 3 | 3 |
| Tharaka-Nithi | 2018 | 387 | 75 | 59 | 0 | 0 |
| Trans nzoia | 2018 | 65 | 8 | 8 | 19 | 7 |
| Turkana | 2018 | 911 | 0 | 0 | 103 | 80 |
| West Pokot | 2018 | 640 | 61 | 23 | 3 | 1 |
| Embu | 2019 | 6 | 6 | 6 | 0 | 0 |
| Garissa | 2019 | 887 | 423 | 345 | 120 | 90 |
| Kajiado | 2019 | 723 | 54 | 42 | 89 | 77 |
| Kisumu | 2019 | 46 | 0 | 0 | 0 | 0 |
| Machakos | 2019 | 420 | 20 | 13 | 30 | 26 |
| Makueni | 2019 | 5 | 0 | 0 | 0 | 0 |
| Mandera | 2019 | 352 | 21 | 21 | 0 | 0 |
| Mombasa | 2019 | 80 | 68 | 25 | 0 | 0 |
| Nairobi | 2019 | 606 | 4 | 4 | 0 | 0 |
| Narok | 2019 | 143 | 6 | 5 | 1 | 1 |
| Turkana | 2019 | 222 | 170 | 169 | 41 | 40 |
| Wajir | 2019 | 674 | 567 | 554 | 6 | 6 |
| Garissa | 2020 | 55 | 7 | 7 | 0 | 0 |
| Marsabit | 2020 | 365 | 27 | 27 | 0 | 0 |
| Murang'a | 2020 | 8 | 0 | 0 | 0 | 0 |
| Turkana | 2020 | 279 | 54 | 51 | 9 | 7 |
| Wajir | 2020 | 4 | 3 | 3 | 0 | 0 |
