# Supplementary material for: Prioritizing interventions for cholera control in Kenya, 2015–2020
Source: PLoS Negl Trop Dis. 2023 May 17;17(5):e0010928. doi: 10.1371/journal.pntd.0010928 (PMC10228803; doi:10.1371/journal.pntd.0010928)
Supplement: Attachment — Submitted filename: Prioritizing interventions for cholera control in Kenya 2015-2020_APK Comments.docx [file pntd.0010928.s003.docx]

**General comments:**

This is a very relevant and important topic, especially with the concern of climate change, waterborne diseases, lac of access to water and sanitation and poor reporting and surveillance system when it comes to cholera control. While you implied in the paper, I think your recommendations would be much stronger if you state 2-3 recommendations, by number (to draw attention), and specific what need to be done in Kenya to improve cholera control and interventions based on your findings.

Intext citations inconsistent. Some have comas and some do not. See examples below.

45 Cholera remains a public health threat to over one billion people globally,^1 2 3^ and the majority.

48 few years, with the most recent wave of outbreaks beginning in December 2014.^5 6,7^

**Specific comments**

24-25 …*.*need to target multi-sectoral interventions to priority areas known as “cholera burden hotspots.” Priorities areas

Suggestions: change statement to prioritize cholera burden hotspot areas

54-56 To combat cholera in Kenya, the Ministry of Health (MoH) developed a national cholera control plan based on the guidance from the WHO-led Global Task Force on Cholera Control (GTFCC). The GTFCC’s *Global Roadmap for Ending Cholera by 2030*^9^

Comment: leave space between 2030 and 9

72-74 We examined all suspected cholera cases across Kenya from the national line list data from January 1, 2015 through July 29th, 2020; no cholera cases were reported after July 2020 so we assumed this to be a complete year of 2020 data.

Comment: It is not clear why the author assumed complete year of data just because data was not reported. The author should just state the period of the data analyzed.

74-75 we assumed this to be a complete year of 2020 data. The study team cleaned location names and performed name disambiguation to match sub-county names to GADM sub-county locations.

Comment: Please spell out GADM and briefly mention what is GADM for clarity.

142-143 When performing the same prioritization analysis at the county scale instead of the sub-county scale, classification becomes much less efficient (Figure 4).

Comment: was this due to low resolution? it would help to simply state at the beginning why classification becomes much less efficient and then go on further and explain. This would provide clarity.

151 sub-counties would be classified as living in low priority counties, there were would be significant

Comment: .... there would be a significant …

152 “over-prioritization” of populations living in sub-counties with no or few cases reported.

Comment: “over-prioritizations” of populations living in sub-counties with no or few cases reported.

181-187 According to the 2009 census, while ~90% of urban households have access to improved water sources, only approximately 50% of rural households have such access^.20^ In addition, the type of improved water source greatly differs, with urban households predominantly having piped water into the dwelling while rural households primarily have access to dug wells as an improved water source. Access to adequate sanitation is also a significant issue in Kenya, with the 2009 census reporting that only 30% of urban and 20% of rural households have access to an improved toilet facility.^20^

Comment: In your statement you state that over 90% have access to improved drinking water sources compared to 50 % in rural areas. It would help to include population size to provide context, and to remind the reader here which one of the counties are considered rural and urban. This would provide perspective and help to understand risk factor especially since you do state that Kenya is one among top 10 countries with largest population without access to improved water sources. Otherwise, 90% of the urban population having access seems pretty good.

*“ 187 – 189 Per UNICEF’s 2012 Update on the Progress of Drinking Water and Sanitation, Kenya is among ten countries with the largest population without access to an improved drinking water source”.^21^*

192-192 The current GTFCC method for priority intervention areas recommends that cholera data are collated at an administrative level 2 or smaller level to facilitate operational planning

Comment: please briefly mention what is level 2 (or remind the reader again, if mentioned previously).

200-201 A recent study focusing on hotspots in Nigeria concluded that targeting locations at the ward level (finer geographic scale)

Comment: to provide perspective, mention if ward would be smaller than sub-county and even go a step further and provide the population size difference between the two so the reader can understand why this approach would be more feasible.

202- 204 was recommended over hotspots at the local government level.^22^ Local knowledge (opinion) was used to focus OCV in specific sub-counties of the districts identified as high risk. This study in Kenya attempts to identify these high-risk sub-counties more objectively.

Comment: please just state and cite or if the opinion is from the author, provide it as a recommendation based on your findings. The way it is written, it is not clear whose opinion it is.
