# Supplementary material for: Prioritizing interventions for cholera control in Kenya, 2015–2020
Source: PLoS Negl Trop Dis. 2023 May 17;17(5):e0010928. doi: 10.1371/journal.pntd.0010928 (PMC10228803; doi:10.1371/journal.pntd.0010928)
Supplement: Attachment — Submitted filename: Response to Reviewers_3.17.23.docx [file pntd.0010928.s004.docx]

**General comments:**

1. This is a very relevant and important topic, especially with the concern of climate change, waterborne diseases, lack of access to water and sanitation and poor reporting and surveillance system when it comes to cholera control. While you implied in the paper, I think your recommendations would be much stronger if you state 2-3 recommendations, by number (to draw attention), and specific what need to be done in Kenya to improve cholera control and interventions based on your findings.
   a. ***Response:***

*Based on the findings from the study we make the following recommends*

- *Cholera hotspot mapping to be used to guide prioritization of specific cholera burden sites targeted for intervention*
- *Need to conduct situation analysis on contextual factors driving cholera outbreak in the targeted sites to inform intervention*
- *Need to digitize cholera surveillance tools including monitoring of WASH inidicators*

*Based on these recommendations, the text on line 257 has been modified to state: “It is the hope that the cholera hotspots mapping results support the MOH Kenya to efficiently utilize available resources through an evidence-based approach to prioritize high risk sub-counties in the implementation of the available interventions. Further, the results emphasize the need to conduct situation specific analyses to understand contextual factors driving cholera outbreaks to inform interventions as well as highlights the need to digitize cholera surveillance tools, including case reporting and monitoring of WASH indicators.”*

2. Intext citations inconsistent. Some have comas and some do not. See examples below.

45 Cholera remains a public health threat to over one billion people globally,^1 2 3^ and the majority.

48 few years, with the most recent wave of outbreaks beginning in December 2014.^5 6,7^

a. ***Response:*** This has been corrected.

**Specific comments**

3. 24-25 …*.*need to target multi-sectoral interventions to priority areas known as “cholera burden hotspots.” Priorities areas

Suggestions: change statement to prioritize cholera burden hotspot areas

a. ***Response:*** *The statement on Line 24 was modified to state “which emphasizes the need to target multi-sectoral interventions in priority cholera burden hotspots”.*

4. 54-56 To combat cholera in Kenya, the Ministry of Health (MoH) developed a national cholera control plan based on the guidance from the WHO-led Global Task Force on Cholera Control (GTFCC). The GTFCC’s *Global Roadmap for Ending Cholera by 2030*^9^

Comment: leave space between 2030 and 9

a. ***Response:*** This has been corrected.
5. -74 We examined all suspected cholera cases across Kenya from the national line list data from January 1, 2015 through July 29th, 2020; no cholera cases were reported after July 2020 so we assumed this to be a complete year of 2020 data.

Comment: It is not clear why the author assumed complete year of data just because data was not reported. The author should just state the period of the data analyzed.

a. ***Response:*** *Our knowledge of the country context suggested that cases were not being reported in the national line list because there were no cases to report for the remainder of 2020. We clarified the analysis period by saying: “We examined all suspected cholera cases across Kenya from the national line list data from January 1, 2015 through Dec 31, 2020”*

6. 74-75 we assumed this to be a complete year of 2020 data. The study team cleaned location names and performed name disambiguation to match sub-county names to GADM sub-county locations.

Comment: Please spell out GADM and briefly mention what is GADM for clarity.

a. ***Response:*** *We explained the abbreviation on line 81 “The* *study team cleaned location names and performed name disambiguation to match sub-county names to Global Administrative Areas (GADM) database of worldwide shapefiles sub-county locations*.”

7. 142-143 When performing the same prioritization analysis at the county scale instead of the sub-county scale, classification becomes much less efficient (Figure 4).

Comment: was this due to low resolution? it would help to simply state at the beginning why classification becomes much less efficient and then go on further and explain. This would provide clarity.

a. ***Response*:** *The sentence has been modified to state “becomes much less efficient due to reduced resolution.”*

8. 151 sub-counties would be classified as living in low priority counties, there were would be significant

Comment: .... there would be a significant …

a. ***Response:*** *This has been corrected.*
9. 152 “over-prioritization” of populations living in sub-counties with no or few cases reported.

Comment: “over-prioritizations” of populations living in sub-counties with no or few cases reported.

a. ***Response:*** *This has been corrected.*
10. 181-187 According to the 2009 census, while ~90% of urban households have access to improved water sources, only approximately 50% of rural households have such access^.20^ In addition, the type of improved water source greatly differs, with urban households predominantly having piped water into the dwelling while rural households primarily have access to dug wells as an improved water source. Access to adequate sanitation is also a significant issue in Kenya, with the 2009 census reporting that only 30% of urban and 20% of rural households have access to an improved toilet facility.^20^

Comment: In your statement you state that over 90% have access to improved drinking water sources compared to 50 % in rural areas. It would help to include population size to provide context, and to remind the reader here which one of the counties are considered rural and urban. This would provide perspective and help to understand risk factor especially since you do state that Kenya is one among top 10 countries with largest population without access to improved water sources. Otherwise, 90% of the urban population having access seems pretty good.

*“ 187 – 189 Per UNICEF’s 2012 Update on the Progress of Drinking Water and Sanitation, Kenya is among ten countries with the largest population without access to an improved drinking water source”.^21^*

a. ***Response:*** *Thank you for this comment, we agree it is critically important to disaggregate water data not just by access but also context. We updated the statement according to the 2021 JMP progress on SDGs, which finds nearly the same percentage of persons with access in rural versus urban households as was recorded in the 2009 census over a decade prior. While the 2021 data confirms there has not been significant change, we did remove the 2012 UNICEF Report that kenya is among the top 10 countries as this is no longer in the 2021 UNICEF report. Additionally, we did not include an analysis the breakdown of the population of each counties urban and rural sub-areas. But to address the question about how vast the rural population is, we included a breakdown of rural versus urban nationally within Kenya, taken from the recent 2019 census. The census also does not offer the breakdown of rural v. urban at a sub-county level, however, nationally it is quite clear that the majority of the population is rural, and therefore lacking the improved water and sanitation. The text now states:*

*“According to the 2009 census, and more recently the 2021 Progress on Household Drinking Water, Sanitation and Hygiene produced by the JMP, ~90% of urban households have access to improved water sources, while only approximately 50% of rural households have such access.^20,21^ In addition, the type of improved water source greatly differs, with urban households predominantly having piped water into the dwelling while rural households primarily have access to dug wells as an improved water source. Access to adequate sanitation is also a significant issue in Kenya, with the 2009 census reporting that only 30% of urban and 20% of rural households have access to an improved toilet facility.^20^ The rural population of Kenya comprises 68.8%, or 32,732,596 people, out of the total population of just over 47million people.^23^”*

11. 192-192 The current GTFCC method for priority intervention areas recommends that cholera data are collated at an administrative level 2 or smaller level to facilitate operational planning

Comment: please briefly mention what is level 2 (or remind the reader again, if mentioned previously).

a. ***Response:*** *The name of administrative level 2 units varies by nation, which is why the generic term is used. We added a parenthetical clarification that this typically means “district” and the following sentence mentions that the analysis for Kenya was performed at the sub-county level, which in Kenya is admin level 3.*

*The text has been reworded to state “The current GTFCC method for priority intervention areas recommends that cholera data are collated at an administrative level 2 (e.g. districts) or smaller level to facilitate operational planning. At the request of the MoH, this analysis was performed at the sub-county level (admin level 3 in Kenya as county is admin level 2)*.”

12. 200-201 A recent study focusing on hotspots in Nigeria concluded that targeting locations at the ward level (finer geographic scale)

Comment: to provide perspective, mention if ward would be smaller than sub-county and even go a step further and provide the population size difference between the two so the reader can understand why this approach would be more feasible.

a. ***Response:*** *Administrative units vary by nation, in Nigeria they do not have counties or sub-counties as they do in Kenya, they have Regional Zones, States, LGAs or wards. However, a ward is an administrative unit level 4. We will include the administrative level to clearly demonstrate that a ward would be smaller than a sub-county. The statement now reads: “A recent study focusing on hotspots in Nigeria concluded that targeting locations at the ward level (administrative unit level 4, which is a finer geographic scale than a sub-county) was recommended over hotspots at the local government area (LGA) level, which is an administrative unit level 3.”*

13. 202- 204 was recommended over hotspots at the local government level.^22^ Local knowledge (opinion) was used to focus OCV in specific sub-counties of the districts identified as high risk. This study in Kenya attempts to identify these high-risk sub-counties more objectively.

Comment: please just state and cite or if the opinion is from the author, provide it as a recommendation based on your findings. The way it is written, it is not clear whose opinion it is.

a. ***Response:*** *We agree that these sentences were unclear. This section has now been revised to clarify the reference to two studies:*

*“In Uganda, the hotspot analysis identified districts as hotspots, but based on local knowledge of the county officials, specific sub-counties were identified as the areas at highest risk and these sub-counties were then targeted for OCV.^1^ Similarly, an analysis of hotspots in Nigeria showed that within the LGAs (Local Government Area) (analogous to a district), certain wards were at higher risk than other wards.^2^	“*

Reviewer's Responses to Questions

 Reviewer #1: The reviewer found the objective of the study to be clear.  There were a few issues that would improve the readers understanding of the methods.

14. The IDSR definition of suspect cholera (3 or more episodes of watery diarrhea in 24 hours, age>2) appears to have low specificity.  ETEC, Campy, Shigella infections would also cause 3 or more episodes in 24 hours for children and adults.  Can the authors provide data supporting the validity of this definition for case detection?  Was there a requirement that suspects cases be epidemiologically linked to a laboratory confirmed case?
a. ***Response:*** *The IDSR nor WHO case definitions for suspect cholera require laboratory confirmation or epidemiological links to a confirmed case (*[*https://www.who.int/emergencies/outbreak-toolkit/disease-outbreak-toolboxes/cholera-outbreak-toolbox#:~:text=WHO%20surveillance%20case%20definition,1%20week%20of%20one%20another*](https://www.who.int/emergencies/outbreak-toolkit/disease-outbreak-toolboxes/cholera-outbreak-toolbox#:~:text=WHO%20surveillance%20case%20definition,1%20week%20of%20one%20another)*). When cholera has been confirmed in a nearby area, the definition of a suspect case no longer has a requirement to be >2 years of age. Unfortunately, systematic testing of suspect cholera is quite rare in most cholera surveillance settings, and we discuss the limitations of the clinical case definition in paragraph 4 of the Discussion.*
15. Provide additional information on how the 90th percentile for MAI was chosen.  Did you rank all MAI by county and select the 90th percentile value? Provide methods for selecting the persistence cutoff too.
a. ***Response:*** *MAI and persistence thresholds were selected after comparing multiple combinations of potential thresholds and consulting with country officials about the relative importance of each metric and the desired size of the populations to be designated as “high priority” for disease control measures. We have modified the following sentence in the methods:*

“*Based on discussions with the MoH regarding the feasibility, cost, and logistics for targeting interventions across the country and the relative importance of each metric to country priorities, we selected the 90th percentile value as the MAI threshold (33 per 100,000) and the median value as the persistence threshold (2.88%) at the sub-county scale.”*

16. For readers unfamiliar with Kenya, it would be helpful to describe the administrative division of the country.  What is the number of counties and subcounties and number rural and urban
    counties/subcounties. How do counties/subcounties differ by population size.  It may be helpful to give an example of a county with total number of subcounties.
a. ***Response:*** *In line 84, we added the following sentence:*

*“In Kenya, there are 47 counties, with an average population size of 1.1 million in 2019, and 301 sub-counties, with an average population size of 176,000 in 2019.”*

17. In figures, it was unclear what the denominator was for "Total Annual Incidence Rate."  Was this the number of cases or case number per population value (e.g., 100,000 persons)?
a. ***Response:*** *Yes, the indicator is cases per 100,000 persons. In the methods line 110 we state:*

*“1) mean annual incidence (MAI), the mean of the annual cholera incidence rate (cases per 100,000 population) over the analysis period”*

18. The authors identified both suspect and confirmed cholera cases.  Can the authors provide data on the frequency of laboratory confirmed cases in high incidence areas?
a. ***Response:*** *We have created supp Table 6 to provide the data available on laboratory confirmation at a county level. We have added this wording to line 177.*

“*In the county-level analysis, only 4,025 out of 29,082 suspected cases (14%) were tested for culture, among which 2,525 (63%) were positive; only 3,327 RDTs were performed (11%), among which 82% were RDT-positive. Among the 3 high priority counties, Tana River reported 30% of RDT positivity among those tested (36/120), while Wajir reported a 93% positivity rate among those tested (591/633). All 3 counties reported similar rates of culture positive when comparing across all counties, with Garissa (591/867) and Tana River (11/16) reporting 68% culture positive among those tested and Wajir reporting 74% culture positive (345/461) (Supp Table 6).”*

19. Reviewer #1: This is a very practical study with implications for cholera control in and outside Kenya.  The limitations section was adequate.  In the limitations section, the authors addressed my concern with the cholera case definition.  It would be appropriate to further explore in brief what are the implications for low specificity.  The authors should consider a brief discussion on alternative, if any, to this approach to selecting target areas.  Are there other approaches that an MoH could consider.
    a. ***Response:*** *The Global Task Force on Cholera Control’s Surveillance working group has been working for several years to improve cholera-related case definitions and methods for selecting priority areas for intervention. Given that cholera is only confirmed via laboratory culture in a small percentage of cases, reported cases is currently the most accurate estimate of disease burden. There are currently several endeavors, including funding by GAVI, to financially support the use of cholera diagnostics to improve our understanding of true disease burden, and future guidance (not yet released) is expected to include cholera test positivity as another dimension to consider for prioritization exercises.*

*We added the following sentence to the 5^th^ paragraph of the discussion: “As the use of low-cost and effective rapid diagnostic tests becomes more pervasive in cholera surveillance, it would be valuable to consider cholera test positivity as an additional indicator when prioritizing areas for intervention.”*

20. GADM database was mentioned.  In a sentence, clarify what is the GADM.  Many readers will not know.
    b. ***Response:*** *Please see the response to #6 above where this was addressed.*

Reviewer #3:

21. Line 99: Could you specify who discussions at the MOH were held with e.g. departments? How many people? And what you mean by “logistics for targeting interventions”? I think it is a very important point that targeting is based on the ability and resources available within the country and this may affect the thresholds set by other countries. For instance, Kenya have said 90th percentile but other settings may need to specify higher or lower based on their capacities. If you could go into 1-2 sentences more detail on the decision making, this would be useful.
a. ***Response:*** *Please see the response to question #15 above*
22. Table 1: is there a full list of the sub-counties included in the high, medium and low priority levels? I would find this useful as a planning tool.
    a. ***Response:*** *Thank you for this comment. We provide the cleaned raw data from the analysis and can provide the subcounty lists by priority in the supplemental files, supp table 5.*
23. Although the authors have talked about how counties and sub-counties would be prioritised during 2015-2020, based on the data available, it would be useful to see what the authors consider recommendations going forward. Should these 13 priority areas from 8 counties be prioritised from 2020 onwards? Could the authors add more to the discussion on what the implications are for control measures? Did the authors re-consult the MoH on what this means for Kenya’s cholera strategy?
a. ***Response:*** *Thank you for this important point. Hotspot mapping captures a cross sectional period of data and is roughly recommended by GTFCC to be updated every five years. Due to the time it takes for interventions to be planned and implemented, it is expected that the outcomes of these analyses will support mid-to-long-term preventive planning, as opposed to short-term emergency response. Our the hotspot mapping in Kenya has significantly contributed to the preparation of a national cholera control plan by the MOH. The hotspot mapping report helped in guiding prioritization of intervention in the high risks sub counties identified and will further help in prioritization of the available resources which can be used to address water and sanitation related risk factors for cholera. The study provides an improved understanding of the location and risk of cholera in Kenya.* *We have revised the last paragraph of the discussion to include this information.*
