# Supplementary material for: Prioritizing interventions for cholera control in Kenya, 2015–2020
Source: PLoS Negl Trop Dis. 2023 May 17;17(5):e0010928. doi: 10.1371/journal.pntd.0010928 (PMC10228803; doi:10.1371/journal.pntd.0010928)
Supplement: Attachment — Submitted filename: Second Response 4.18.23.docx [file pntd.0010928.s005.docx]

Dear Dr Debes,

It looks like your submission is currently still in your author account awaiting a few formatting updates requested on March 29 - I see that you sent a query about these requests on April 12, and I sincerely apologize if you did not receive a response. The changes in question are as follows:

1) In the submission form, on the author entry page, please enter CRediT contributions for co-author Daniel Langat (the contribution options are listed here: <https://journals.plos.org/plosntds/s/authorship#loc-author-contributions>).

Response: This oversight has been corrected. Apologies.

2) The file inventory includes files for Figures 3a, 3b, and 3c. We would recommend either combining these into a single Figure 3 .tiff file with separate internal panels, or renumbering them as individual figures (Figures 3, 4, and 5), as we are not able to publish multiple components of a single figure as separate files. (It appears that 'Figure 3' is cited individually within the manuscript, so combining the panels into a single Figure 3 file may be the easiest option).

Response: The figures were separated as suggested to Figure 3, 4 and 5. Prior Figure 4 was moved to Figure 6 and the manuscript updated. TC submitted.

Once you have made these changes to the submission form and file inventory, your paper will be returned to the Section Editor for re-evaluation.

Kind regards,

Sarah
